# Comprehensive molecular characterization of microneedling therapy in a human three‐dimensional skin model

**DOI:** 10.1371/journal.pone.0204318

**Published:** 2018-09-20

**Authors:** Laurenz Schmitt, Yvonne Marquardt, Philipp Amann, Ruth Heise, Laura Huth, Sylvia Wagner-Schiffler, Sebastian Huth, Jens-Malte Baron

**Affiliations:** 1 Department of Dermatology and Allergology, Medical Faculty, RWTH Aachen University, Aachen, Germany; 2 Praxis für Dermatologie, Aachen, Germany; NYU Langone Medical Center, UNITED STATES

## Abstract

**Background and objectives:**

Microneedling therapy is a widely used technique in dermatology. However, little is known about the underlying molecular effects of this therapy on extracellular matrix remodeling, wound healing, and inflammation. The aim of this study was to examine morphological and molecular changes caused by microneedling treatment in a standardized *in vitro* full-thickness 3D model of human skin.

**Materials and methods:**

A microneedling device was used to treat full-thickness 3D skin models. Specimens were harvested at specified time points and qRT-PCR and microarray studies were performed. Frozen sections were examined histologically.

**Results:**

Microneedling treatment caused morphological changes in the skin model resulting in an almost complete recovery of the epidermis five days after treatment. Microarray analysis identified an upregulation of genes that are associated with tissue remodeling and wound healing (e.g. *COL3A1*, *COL8A1*, *TIMP3*), epithelial proliferation and differentiation (*KRT13*, *IGF1*), immune cell recruitment (*CCL11*), and a member of the heat shock protein family (*HSPB6*). On the other hand, we detected a downregulation of pro-inflammatory cytokines (e.g. *IL1α*, *IL1β*, *IL24*, *IL36γ*, *IL36RN*), and antimicrobial peptides (e.g. *S100A7A*, *DEFB4*). These data were confirmed by independent RT-PCR analyses.

**Conclusion:**

We present for the first time the direct molecular effects of microneedling therapy on epidermal keratinocytes and dermal fibroblasts using a standardized 3D skin model. Treatment resulted in histological alterations and changed the expression of various genes related to epidermal differentiation, inflammation, and dermal remodeling. This data suggests that skin microneedling plays a role in dermal remodeling, increases epidermal differentiation, and might also have a direct effect on collagen synthesis. These findings may increase our understanding of the molecular mechanisms of human skin repair induced by microneedling therapy and will allow comparisons with competing applications, such as ablative laser therapies.

## Introduction

Skin microneedling therapies are growing in popularity for the treatment of a wide variety of dermatological conditions [1]. This technique has most commonly been used to treat atrophic acne scars [2], striae distensae [3], melasma [4], and to promote skin rejuvenation [5]. Skin microneedling is also used in combination with skin cell transplantation to treat burn victims and for transdermal drug delivery [6–8]. Due to its low cost and easy handling compared to ablative and non-ablative laser therapies, this method is becoming increasingly popular and research in this field recently intensified [9, 10]. Skin treatment procedures vary broadly from chemical techniques, to laser treatments, to surgical interventions [11]. However, most of these treatments are invasive and can cause secondary problems like hyper- or hypopigmentation, especially in patients with darker skin types [12]. Clinical results for potential skin rejuvenation showed promising results with microneedling (1–1.5 mm needle length) similar to those with medical needling (3 mm needle length) [10]. A major advantage of microneedling is that it is less invasive and can be applied under local anesthesia. In microneedling therapy, the epidermis remains relatively intact, which helps to limit post procedural adverse events, such as bleeding, swelling, and pain [13].

Human organotypic 3D skin models have established themselves as a standard method for studying human skin and have revealed interesting and reproducible results after laser induced microwounding [14–17]. We previously developed a standardized human 3D skin model for studying morphological and molecular modifications during wound healing after laser treatment [18]. Until now, little is known about the underlying molecular and histomorphological effects of microneedling treatment on human skin, because changes in the expression of various growth factors (TGFβ1–3, FGF, EGF, VEGF, TNF-α) that promote collagen synthesis have only been described in animal skin biopsies [7]. Therefore, the aim of the present study was to investigate the time-dependent histological and molecular alterations following microneedling treatment in an established human 3D skin model.

## Materials and methods

### Isolation and culture of normal human epidermal keratinocytes (NHEK) and normal human dermal fibroblasts (NHDF)

NHDF and NHEK were isolated from biopsies of four different donors after cutaneous surgery. The epidermis was separated from the dermis by digestion with dispase (BD Biosciences, Franklin Lakes, NY) and trypsin (Lonza, Basel, Switzerland). Trypsin Neutralization Solution (Lonza) was used for pH-neutralization. The dermal portion of the biopsy was incubated in collagenase 1A (Sigma, Taufkirchen, Germany) to yield a single cell suspension of NHDF. This study was conducted according to the Declaration of Helsinki and was approved by the ethics committee of the University Hospital, RWTH Aachen, Germany. Written informed consent was obtained from the skin donor. Cultivation of NHEK and NHDF was performed as described previously [18].

### Scaffold skin equivalents

Matriderm (Medskin Solutions, Suwelack A.G., Billerbeck, Germany) is a 3D bovine collagen–elastin matrix consisting of bovine collagen types I, III, and V. In this study, matrices of 148 x 105 x 1 mm were used. The collagen–elastin matrix was sliced into circular 22 mm punches and transferred into six-well cell culture inserts (BD Falcon, Bedford, MA, USA), then stored under sterile conditions in six-well plates until use. Matriderm scaffolds were inoculated with 3 x 10^5^ NHDF per cm^2^ in Tisseel (Baxter, Derfield, IL, USA) and submersed with fibroblast growth medium used for dermal scaffold skin equivalents. After three days, 3 x 10^6^ NHEK were seeded on top of each dermal equivalent. Skin equivalents were submersed in equal volumes of DMEM and keratinocyte growth medium with 5% fetal calf serum (FCS), 50 μg ascorbic acid, and 5 μg/ml aprotinin (Applichem, Chicago, IL, USA). On the following day, skin equivalents were lifted to the air–liquid interface. The calcium concentration of the culture medium was increased to 1.0 mM and medium was changed every other day [18].

### Skin needling

3D skin models were treated with an eDermastamp (Dermaroller GmbH, Wolfenbüttel, Germany) using a six needle plate (1.5 NM615LS16309, Amiea Med). One hundred insertions were made per second at a penetration depth of 1.0 mm and with three passes, according to the manufacturer´s and clinical treatment recommendations. After treatment, the models were cultivated in fresh culture medium and harvested on day 5 for histological analysis and detection of gene expression. Untreated models were maintained as negative controls. All experiments were performed in triplicate for every time point.

### RNA isolation

Total RNA was isolated using the Nucleo Spin RNA Kit (Macherey and Nagel, Düren, Germany) according to the manufacturer’s instructions. RNA isolation included on-column digestion of DNA with RNase-free DNase I. The RNA was quantified by photometric measurement (NanoDrop Technologies, Wilmington, DE, USA) and its integrity was analyzed on a 2100 bioanalyzer (Agilent Technologies, Palo Alto, CA, USA).

### Quantitative reverse transcription polymerase chain reaction (qRT-PCR)

Purified RNA was reversed transcribed with SS VILO Mastermix (Life Technologies) according the manufacturer’s instructions. TaqMan experiments were carried out on an ABI Prism 7,300 sequence detection system (Applied Biosystems, Weiterstadt, Germany) using Assays-on-Demand gene expression products for *CCL11* (Hs00237013_m1), *IGF1* (Hs01547656_m1), *TIMP3* (Hs00165949_m1), *KRT13* (Hs00357961_g1), *COL3A1* (Hs00943809_m1), *HSPB6* (Hs00328933_m1), *COL8A1* (Hs00156669_m1), *IL1α* (Hs00174092_m1), *IL1β* (Hs00174097_m1), *IL24* (Hs01114274_m1), *IL36γ* (Hs00219742_m1), *IL36RN* (Hs_00202179_m1), *S100A7A* (00752780_s1), and *DEFB4* (Hs00823638_m1), according to the manufacturer’s recommendations. An Assay-on-Demand product for *HPRT* (Hs99999909) was used as an internal reference to normalize the target transcripts. All measurements were performed in triplicate in separate reaction wells.

### Gene expression analysis using exon expression arrays

Purified mRNA was analyzed on GeneChip Human Gene 2.0 ST arrays as reported previously [18] using Gene-SpringGX software, version 14.9 (Agilent Technologies, Frankfurt am Main, Germany). Gene ontology (GO) analysis was performed using http://www.gene-ontology.org/.

### Light microscopy

For light microscopy, 4 μm cryosections of skin equivalents were embedded in Tissue Tec OCT and stained with hematoxylin and eosin. Sections were examined by a photomicroscope (DMIL, Leitz, Wetzlar, Germany).

### Statistical analysis

Data are given as arithmetical means ± standard deviation and were analyzed with the Mann-Whitney U test using GraphPad PRISM, version 7 (La Jolla, CA, USA). P values <0.05 were considered statistically significant.

## Results

**Fig 1:**
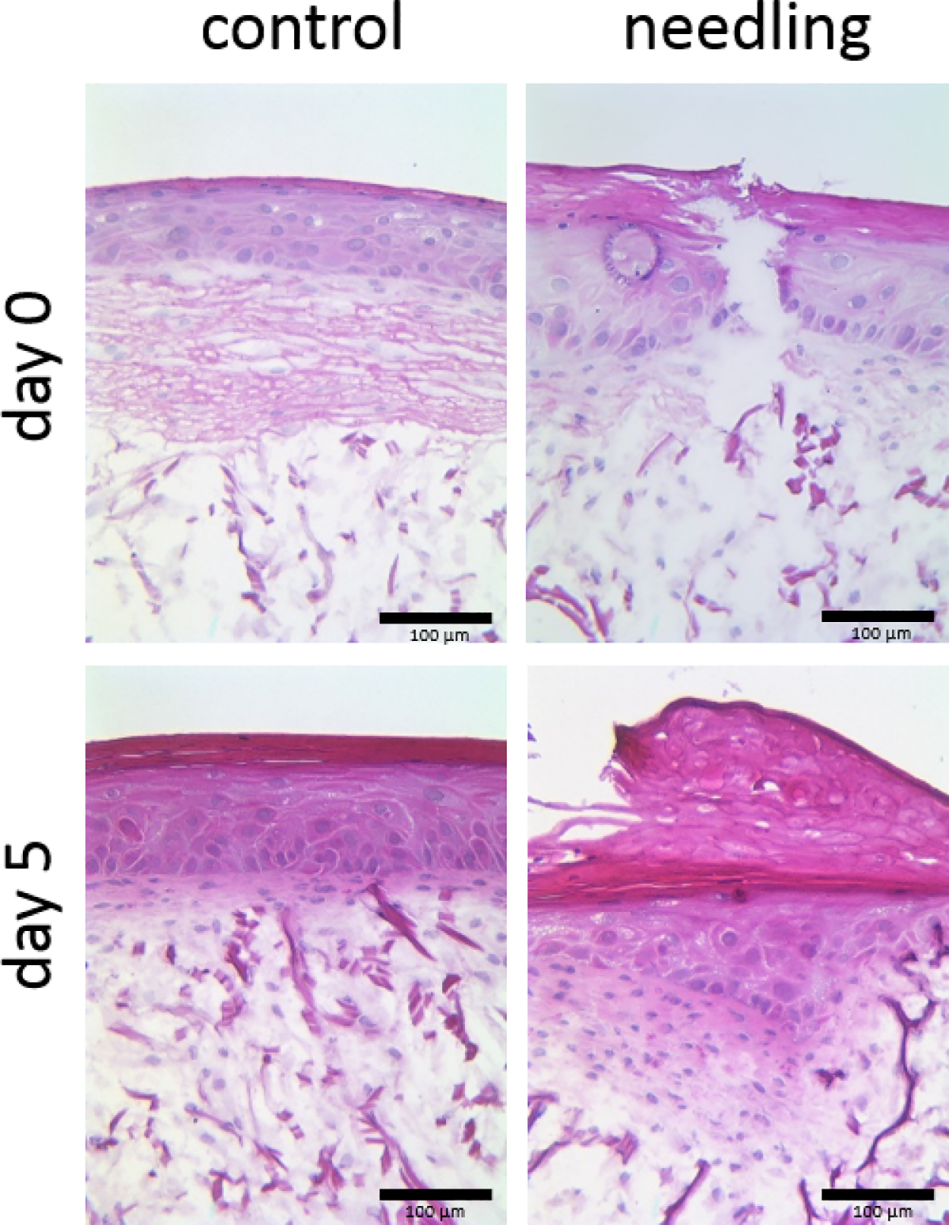
Representative hematoxylin and eosin stained sections of 3D skin models zero and five days after microneedling treatment. Untreated models are shown as controls. Magnification = 100 x, scale bar = 100 μm.

To investigate the effects of microneedling therapy on skin morphology, we established full-thickness human 3D skin equivalents containing dermal and epidermal structures, including a functional stratum corneum, a basal layer, and a basal membrane. Fig 1 depicts representative images of 3D skin models directly after microneedling and five days later compared with untreated controls. Histological examination revealed clearly defined lesions of the epidermis and dermis immediately after microneedling treatment, whereas dermal and epidermal structures were almost totally restored after five days.

**Fig 2:**
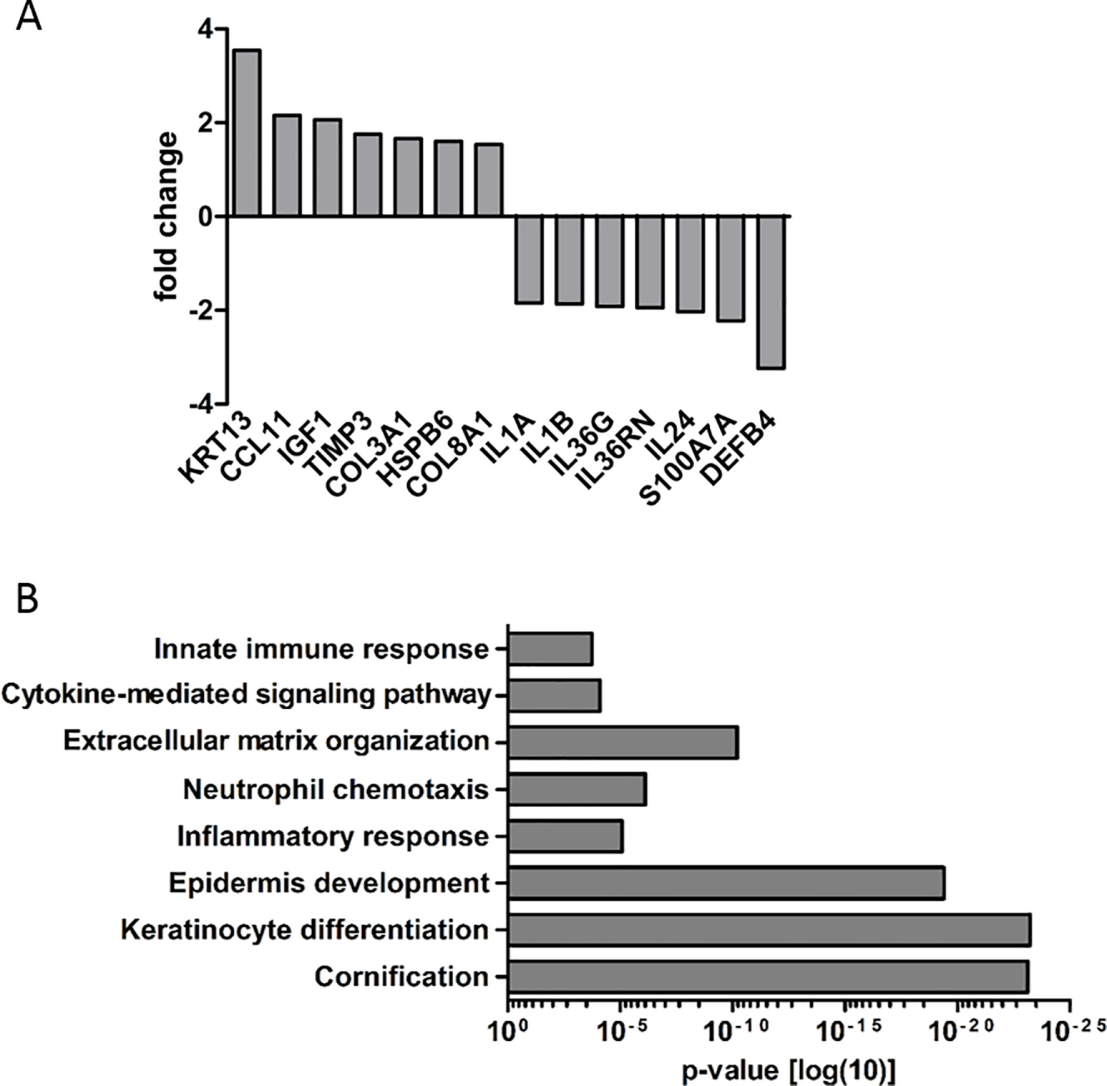
Gene expression profiling in microneedling-treated 3D skin models (microarray analysis). (A) 3D skin models were harvested five days after microneedling treatment. Gene expression was measured using the Affymetrix Gene Chip Human Exon 2.0 ST array. (B) Gene ontology (GO) analysis of microarray results.

**Fig 3:**
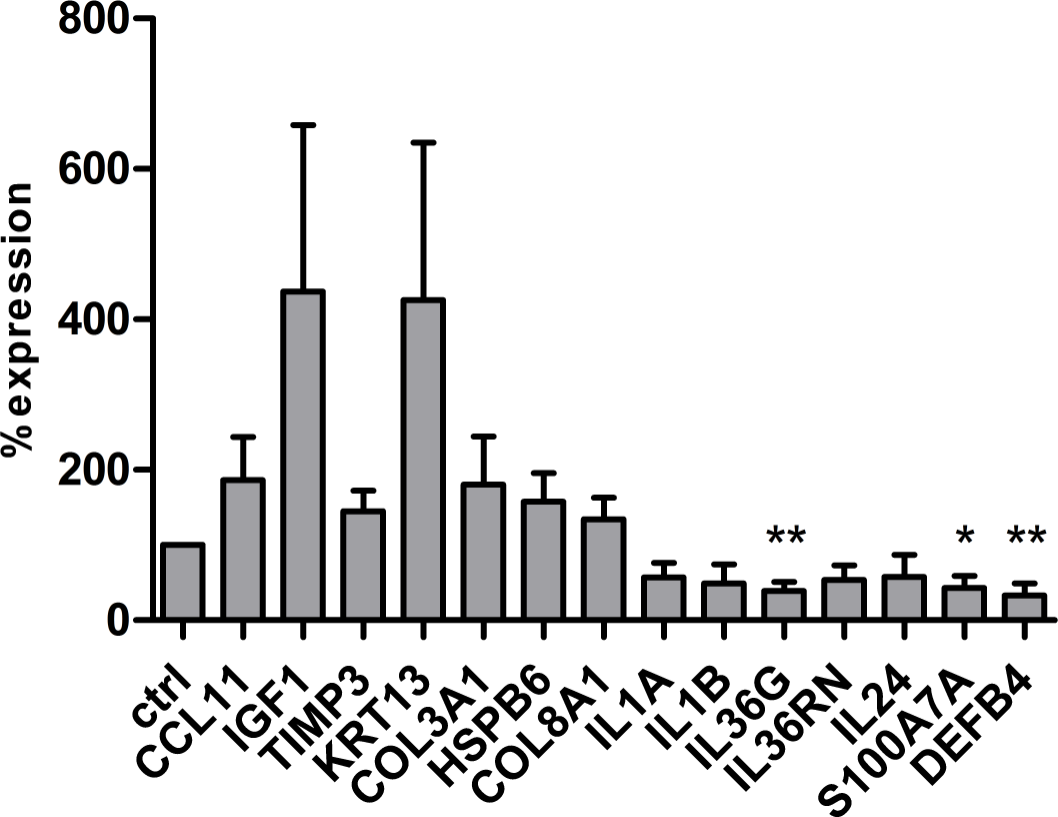
TaqMan real-time PCR analysis of four independent approaches displaying the expression of selected genes five days after microneedling treatment.Ctrl = control. *p < 0.05, **p < 0.01.

To our knowledge, the molecular effects of microneedling on human skin are poorly defined. In the present study, we conducted a transcriptomic microarray profiling of 3D skin models (n = 3) five days after microneedling (Fig 2A). Gene array analysis revealed a >1.5-fold upregulation of genes associated with tissue remodeling and wound healing (*TIMP3*, *COL3A1*, *COL8A1*), epithelial proliferation and differentiation (*KRT13*, *IGF1*), immune cell recruitment (*CCL11*) and an upregulation of heat shock protein (HSP) B6 in microneedling-treated skin models compared with untreated controls. On the other hand, we detected a downregulation of different cytokines (*IL1A*, *IL1B*, *IL36G*, *IL36RN*, *IL24*) as well as antimicrobial peptides (*S100A7A*, *DEFB4*) in microneedling-treated skin models compared with untreated controls. Additionally, GO analysis confirmed an impact of microneedling treatment on biological processes such as “cornification”, “keratinocyte differentiation”, “epidermis development”, “inflammatory response”, and “extracellular matrix organization” (Fig 2B). To verify these findings, we used RT-PCR analysis to measure the expression of selected genes in four independent approaches (Fig 3). In general, RT-PCR analysis confirmed up- and downregulation of all genes. The downregulation of *IL36G*, *S100A7A*, and *DEFB4* in microneedling-treated models was particularly significant.

## Discussion

Skin microneedling has been used to successfully treat acne scars [2] and striae distensae [3], and to promote skin rejuvenation [8]. It has also been used in combination with skin cell transplantation, in transdermal drug delivery, and during pretreatment with photodynamic therapy (PDT) [6, 7, 19]. In a case of melasma, upper dermal neocollagenesis, restoration of basal membrane, and epithelial acanthosis were observed after two sessions of gentle microneedling, reinforcing the hypothesis that microneedling can induce repair [20]. This evolving technique is considered minimally invasive with low costs and a high safety profile [9]. Nevertheless, little is known about the underlying molecular effects of microneedling therapy on the skin.

So far, microneedling has been clinically evaluated in animal skin [7] or in individual human skin biopsies [21] only revealing changes in growth factor expression associated with the *de novo* synthesis of collagen (e.g. *TGFβ1–3*, *FGF*, *EGF*, *VEGF*, *TNF-α*) [7]. To our knowledge, the present study is the first report of the effects of microneedling therapy in a standardized *in vitro* human 3D skin model. In previous studies, we could reveal that human skin equivalents are a suitable standardized *in vitro* tool for detecting the *ex vivo* effects of various laser systems on skin physiology, skin morphology, and gene regulation [14, 18, 22]. Full-thickness skin models seemed to be suitable for studying the effects of microneedling therapy, particularly on deeper dermal layers. Therefore we applied this model system to systematically analyze time-dependent histological and molecular alterations in the skin following microneedling therapy.

Using microarray and RT-PCR analyses, our findings revealed a downregulation of pro-inflammatory cytokines such as *IL1α*, *IL1β*, *IL24*, *IL36RN*, and *IL36γ* as well as antimicrobial peptides (AMPs) such as *S100A7A* and *DEFB4* in microneedling-treated skin models. The wound healing process consists of an acute inflammatory phase, reepithelization, and remodeling of the dermal extracellular matrix [23]. Initiating and perpetuating the inflammatory response involves many molecules, including cytokines and AMPs [24]. Acute inflammation usually lasts for 2–5 days and ceases once the harmful stimuli have been removed [24], which is consistent with our finding that pro-inflammatory cytokines and AMPs are downregulated in 3D skin models five days after microneedling. This downregulation may act as a wound-related signal to stimulate reepithelization.

On the other hand, we detected an upregulation of the known chemokine eotaxin (CCL11). CCL11 regulates cell activation and contributes to angiogenesis [25]. Overall, changes in interleukin and chemokine expression may modify the formation and structural integrity of the epidermis after needling therapy and thereby contribute to neocollagenesis and wound healing.

Matrix metalloproteinases (MMPs) are involved in the remodeling of abnormal scars and also influence other wound healing responses, such as inflammation and reepithelization [26–28]. Tissue inhibitors of metalloproteinase (TIMPs) are downregulated in hypertrophic scars [29]. Interestingly, we detected an upregulation of TIMP3 after microneedling therapy.

A study by Dohi et al. showed that downregulation of TIMP2 contributes to the progression and development of keloids suggesting that higher levels of TIMPs may be beneficial to the reduction of the thick dermis and collagen bundles seen in keloids [30]. In this context, microneedling induced expression of TIMP3 could suggest a positive effect of this therapy in the treatment of hypertrophic scars.

Furthermore, we detected an upregulation of insulin-like growth factor 1 (*IGF-1*) in our skin model five days after microneedling. Lewis et al. [31] showed that *IGF-1* expression in human dermal fibroblasts is triggered by stress, such as UVB-induced DNA damage, which alters the protective stress response of epidermal keratinocytes. In our study, *IGF-1* expression was upregulated after microneedling treatment. This may represent a response to stress-induced cell damage that induces DNA-repair mechanisms. The highly ordered process of wound healing comprises the coordinated regulation of cell proliferation and migration as well as tissue remodeling. These processes are predominantly regulated by polypeptide growth factors, such as members of the insulin growth factor family [32]. The importance of IGF-1 in wound healing has already been shown in several studies [33, 34]. Our findings are in line with those of studies that show increased IGF-1 expression during wound repair processes and support the hypothesis that IGF-1 signaling is required for efficient re-epithelialization and wound healing [24, 34].

It has already been clinically demonstrated that microneedling can induce collagen synthesis following aesthetic surgery and may represent an alternative to laser surgery [35]. Matrix remodeling following the inflammatory phase of wound healing is characterized by collagen synthesis [36]. In this context, we detected an upregulation in the expression of genes that are related to collagen synthesis (*COL3A1*, *COL8A1*) five days after microneedling.

Moreover, expression of HSPs, such as HSPB6, was higher after microneedling therapy in treated skin models than in untreated controls. HSPs were first described as proteins that protect cells following heat stress-related damage [37, 38]. Since then, it has been hypothesized that HSPs also modulate wound contraction in fibroblast cell lines after wound infliction [39]. Previous studies have shown increased HSP expression after ablative fractional resurfacing treatment, suggesting that the heat shock response was most likely due to the thermal effects of the laser treatment [37, 40]. Beside their protective functions, accumulating evidence indicates that HSPs are also involved in tissue remodeling and wound healing [40–42]. Our findings support the theory that HSPs play an important role in dermal remodeling after microneedling treatment.

Interestingly, we found an increased expression of the known epithelial cell differentiation markers keratin 13 (*KRT13*) after microneedling treatment. Wound healing is not fully completed five days after microneedling; therefore the upregulation of this marker in our model may reflect the ongoing differentiation of epithelial cells.

It is worth mentioning that microneedling treatment in 3D models causes similar changes in the expression of differentiation markers as ablative laser therapy using a Er:YAG laser [22, 43]. This may be explained by the fact that both treatments completely remove the epidermis, which then needs to be fully re-developed. In agreement with the observed changes in gene expression, we found an impact of microneedling therapy on biological processes like “cornification”, “keratinocyte differentiation”, “epidermis development”, “inflammatory response”, and “extracellular matrix organization”. These data support the impact of microneedling on wound healing, re-epithelialization, and skin rejuvenation.

The human 3D model system used in the present study is a useful tool for studying physiology, morphology, and time-dependent gene expression after microneedling treatment. The corium is fully developed and allows the effects of microneedling on the collagen structure in deeper skin layers to be examined. Molecular mechanisms underlying the proliferative effect of e.g. pantothenate or laser treatments were previously investigated by global gene expression analysis (microarray analysis) in cultured human dermal fibroblasts (*in vitro*) and in a clinical trial (*in vivo*) [18, 22, 44]. A limitation of this simplified human *in vitro* 3D skin model, which contains only two cell types (keratinocytes and fibroblasts), is that it is not able to mimic the complex requirements of *in vivo* conditions. However, the advantage is that the observed changes in gene expression can be specifically attributed to changes in keratinocytes and fibroblasts. In summary, our findings have revealed that microneedling therapy in a 3D human skin model induces histological alterations and changes the expression of various genes related to epidermal differentiation, inflammation, and dermal remodeling. Based on our results, we assume that microneedling therapy stimulates collagen synthesis, which may be beneficial for skin rejuvenation or the treatment of atrophic scars. Further in vitro studies with novel 3D skin models which additionally also contain macrophages appear to be useful in order to take into account the indirect effects of the microneedling therapy on inflammatory cells.

## Supporting information

S1 Table Supporting information files. http://www.ncbi.nlm.nih.gov/geo/query/acc.cgi?acc=GSE119425.
(XLS)
